# Dr. Domeier, on the Cure of Polydipsia
*See Medical and Physical Journal, No. xvi. p. 506.


**Published:** 1800-10

**Authors:** W. Domeier

**Affiliations:** London


### *Dr.* Domeier, *in Answer to Dr.* Dyce's *Letter.**

Sir,

I Aſſure you, that it is not without unpleaſant feelings that I undertake to communicate to you publicly, in a language which I am not accuſtomed to write, a caſe like your's, which I treated ſucceſsfully: And I certainly ſhould not expoſe in an unpoliſhed, and even perhaps not faultleſs ſtyle, what were my thoughts on the indiſpoſition in queſtion, if it was not for the ſucceſs I met with during my treatment of it.

It would be a particular ſatisfaction to me, to hear that the propoſed plan was likewiſe to anſwer your wiſhes; I then ſhould be the ſurer of being pardoned for the unphilological way in which I exhibit the ſtory of my patient.

It was at Berlin, in the beginning of May, 1799, when a French clergyman, forty years of age, of a melancholy diſpoſition, and conſtant ſedentary life, came to me for my advice. His complaint was an uncommon thirſt of eighteen months ſtanding; ſince the beginning of which, till then, he had remarked nothing elſe wrong in his conſtitution, but his growing thinner, and in conſequence of it, weaker in bearing bodily fatigue. To his beſt remembrance, he had never had any illneſs in his life; and after a minute examination, I concluded that all his animal functions were in perfect order. His appetite was ſtrong, his tongue clean, and his ſleep good, when not interrupted by the violent thirſt. His melancholy turn of mind he attributed to his preſent ſituation, living but poorly, and far from his own home; but his friends told me that he was, when in France, of the ſame diſpoſition.

When he applied to me, he ſaid he had already been to ſeveral phyſicians, who gave him no medicine, anſwering, that there was no remedy for him; he therefore employed whatever remedies his friends recommended to him, but all proved uſeleſs. The quantity of liquids he ſwallowed was enormous. He could hardly put the glaſs out of his hand, before he vehemently deſired to drink again. He moſtly drank water, for he could afford nothing elſe; but whatever liquid he could get was deſirable, viz. tea, coffee, beer, wine, milk, &c.

The quantity of urine he made, did not, even to the fourth part, correſpond with the quantity he drank; beſides, he eat a great deal of watery nouriſhment, as ſoups, fruits, vegetables, &c.

---

* See Medical and Phyſical Journal, No. xvi. p. 506.

&c. a circumſtance which firſt of all attracted my attention, for this uncommon quantity of fluids ought to remain ſomewhere, as his ſtools were perfectly regular; for great thirſt is a common ſymptom in profuſe evacuations, as in copious diarrhœas, diabetes, and even hæmorrhagies.

Having, therefore, no other probable indication to found the cure upon, I thought that the inſenſible perſpiration was too much increaſed, and that the only plan for a cure would be to check this, if I poſſibly could, and to order the patient a beverage which has the virtue of quenching thirſt in the ſmalleſt quantity.

To anſwer the firſt purpoſe, I deſired the patient to dreſs much cooler than he was accuſtomed to do; to uſe the cold bath, and to dry himſelf very negligently afterwards; to put his feet from time to time into cold water, and keep on his ſtockings for a conſiderable time afterwards; to expoſe himſelf to the cool evening and morning air, often even to a thorough air, and to be very thinly covered in bed. His dinner was to be quite cold; nothing warm was allowed him to drink.

To anſwer the ſecond purpoſe, (it being known that acidulous beverages extinguiſh thirſt much better than any other kind) I thought that a mineral acid would anſwer ſtill better, and in conſequence preſcribed him ſulphuric acid, to be mixed with water to a pleaſant degree of acidity, and to drink of it as much as he pleaſed.

After he had followed this advice with exactneſs about a fortnight, he found himſelf a great deal better; he then had intervals of about an hour, in which he did not deſire to drink at all.

I altered nothing, and he was very willing to continue the ſame plan. After full three weeks, his complaint was entirely gone. However, I thought it prudent to make him continue the ſame regimen for a longer time, and leave it off by degrees; but when he had continued it for a very ſhort time, after his recovery, he thought himſelf in perfect ſecurity, got tired of it, and left off all the preſcriptions at once.

He remained well for a few weeks, when the unnatural thirſt began again; and thinking that this proceeded only from the weather, which had become warmer, he did not immediately apply to me. But the thirſt increaſed, and came to the ſame unpleaſant degree it had done before. He then applied to me again; upon which I ordered him to do juſt the ſame as at firſt. He did, and got well again, and even in a ſhorter time. He continued now for a long while to apply one or the other of the preſcribed remedies, eſpecially the cold bath bath, and remained perfectly well till April, 1800, when I ſaw him laſt.

Now I am perfectly aware that this is only one obſervation, and therefore not to be termed an experience, and that it would be wrong to pretend with certainty that my concluſion was ſure, and that my remedies, in conſequence, removed the diſeaſe; however, there is a great probability in their favour, as both times the indiſpoſition was done away exactly in the ſame manner, and in a ſhort time after the remedies were applied; and beſides, the patient had altered nothing in his manner of living to which the cure could be aſcribed.

It would be a particular ſatisfaction to me if the treatment which ſucceeded with my patient ſhould likewiſe anſwer your wiſhes; and, I dare ſay, it will not be unintereſting to the readers of the Medical Journal, to make it known in this way.

I have the honour to be, Sir,

Your moſt obedient ſervant,

